# Supplementary material for: AXL Knock-Out in SNU475 Hepatocellular Carcinoma Cells Provides Evidence for Lethal Effect Associated with G2 Arrest and Polyploidization
Source: Int J Mol Sci. 2021 Dec 9;22(24):13247. doi: 10.3390/ijms222413247 (PMC8708332; doi:10.3390/ijms222413247)
Supplement: Supplementary file 1 [file ijms-22-13247-s001.zip › ijms-1477839-supplementary.pdf]

Supplementary Tables

Table S1: List of primers and probes used in quantitative real-time PCR

Primers and probes used in quantitative real-time PCR

**AXL FL_forward**	CCTCCCAGTACCCCTGGA	Probe 37
**AXL FL_reverse**	AGGCAGGAGTTGAAGGTTCC	Probe 37
**AXL V_forward**	TGTGTGGCAGCCTACACTG	Probe 1
**AXL V_reverse**	AAGGTTCCTTCACTGGGCG	Probe 1
**GAPDH_forward**	CTCTGCTCCTCCTGTTCGAC	Probe 60
**GAPDH_reverse**	AATCCGTTGACTCCGACCTT	Probe 60

Table S2: List of primers of sgRNAs used in cloning

Sequences of sgRNAs

**hAxl-45F**	**CACCgGTGCTTGGCGCTGTGCGGCT**
**hAxl-45R**	**AAACAGCCGCACAGCGCCAAGCACc**
**hAxl-185F**	**CACCgGAGAGCCCCCCGAGGTACAT**
**hAxl-185R**	**AAACATGTACCTCGGGGGGCTCTCc**
**Ren-208F**	**CACCgGTAGCGCGGTGTATTATACC**
**Ren-208R**	**AAACGGTATAATACACCGCGCTACc**

Supplementary Figures:

A

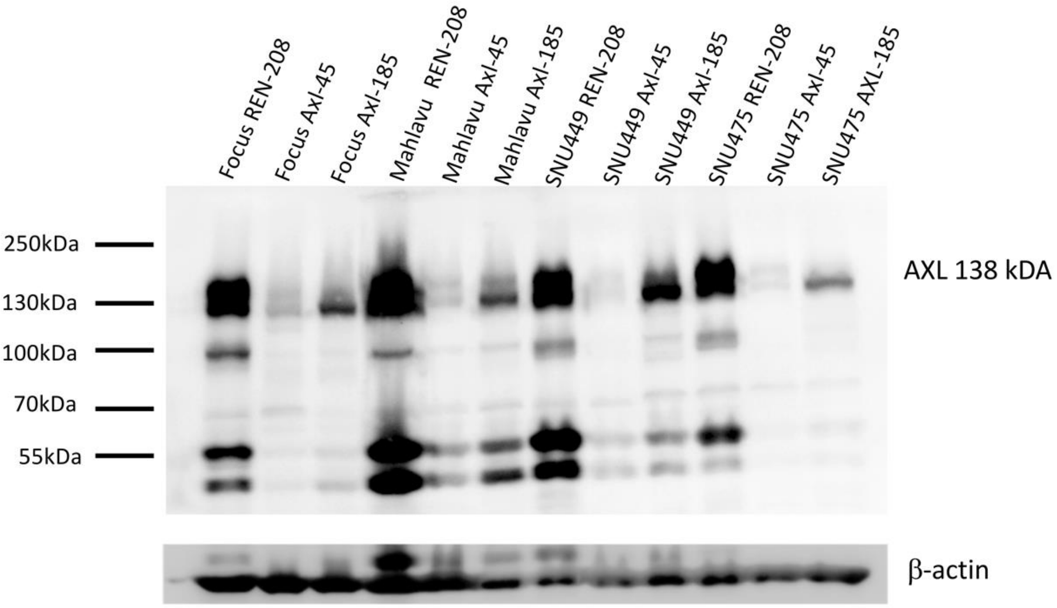

B

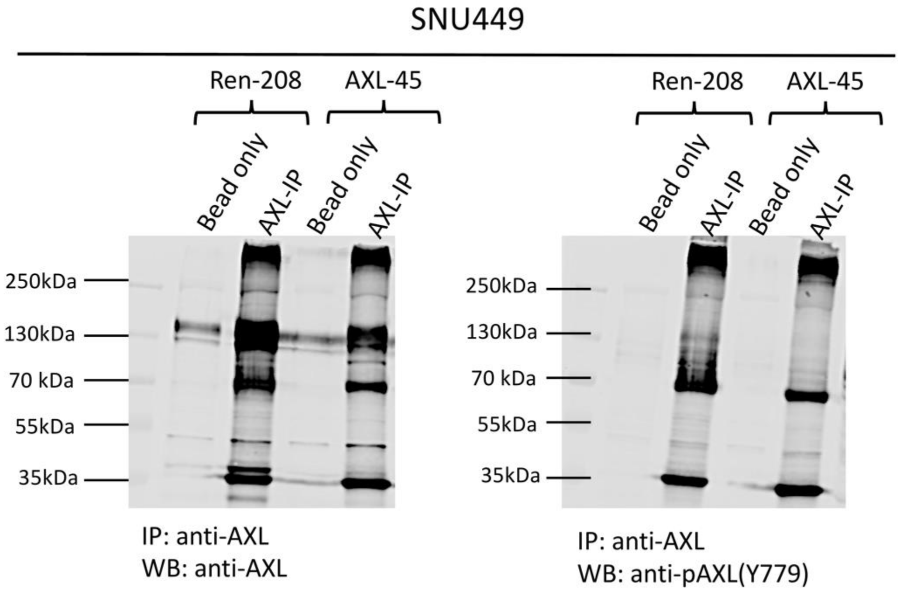

Figure S1. AXL expression of CRISPR-Cas9 mediated knock-out cells. A. Expression of AXL of 4 different HCC cell line Focus, Mahlavu,SNU449 and SNU475 were targeted gRNA REN-208,AXL-45 and AXL-185. B. anti- AXL blot and anti-pAXL(Y779) blot of anti AXL immunoprecipitated SNU449 cells targeted by Ren-208 and AXL-45.

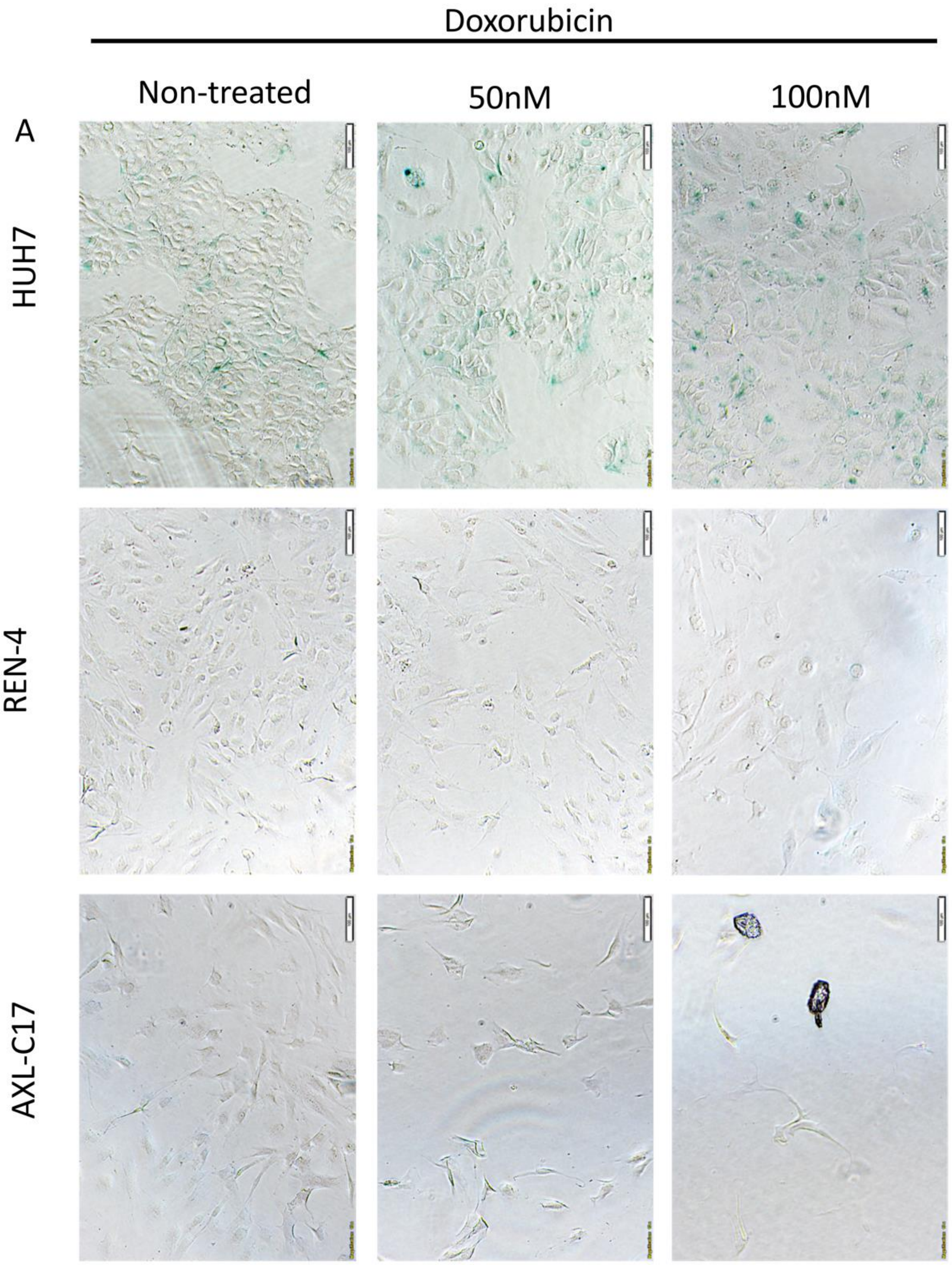

Figure S2. β -Galactosidase Assay of AXL-knockout cells. Huh7 parental was used as positive control. Non-treated, 50 and 100 nM of Doxorubicin were used.

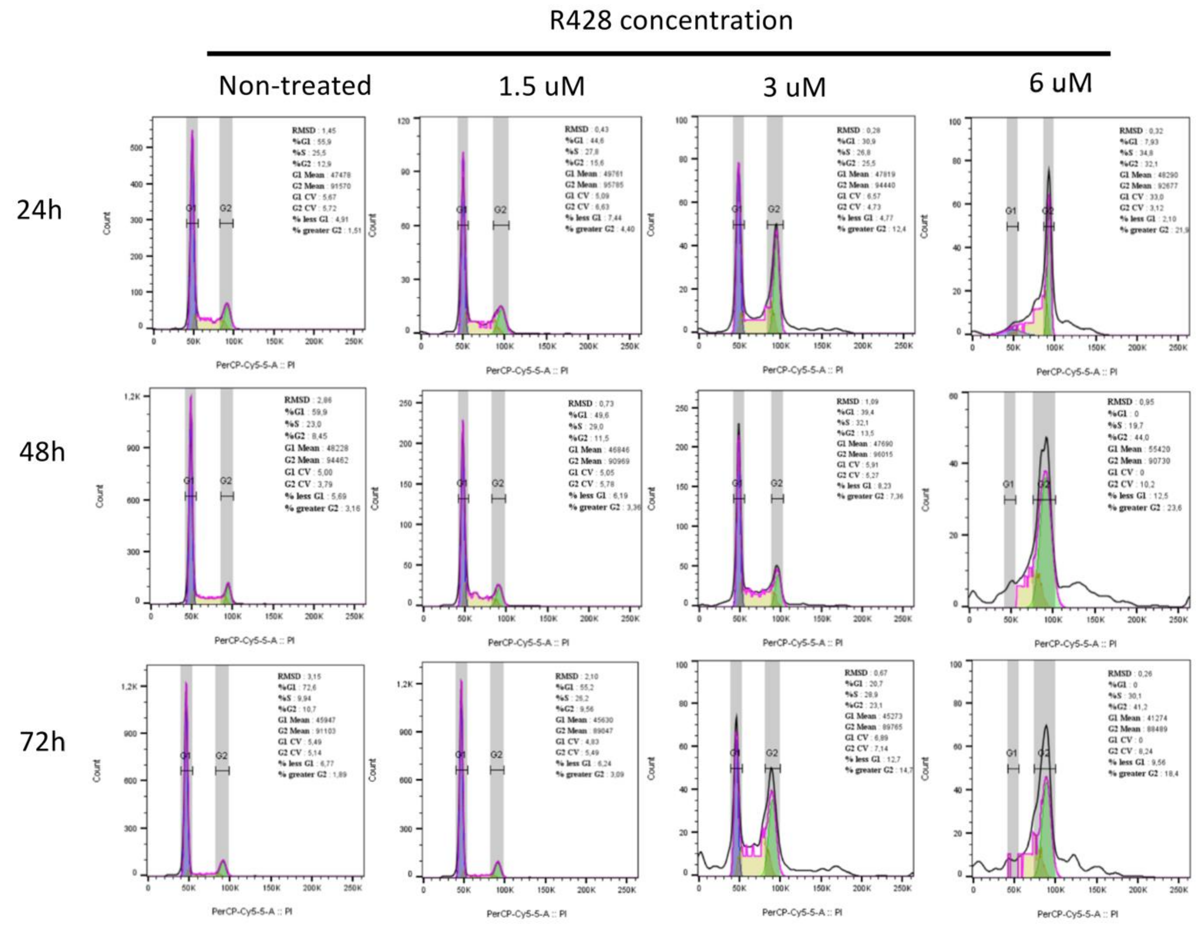

Figure S3. Cell cycle analysis of the parental SNU475 cell line treated with 0,1.5, 3 and 6μM R428 for 24, 48 and 72 hours.

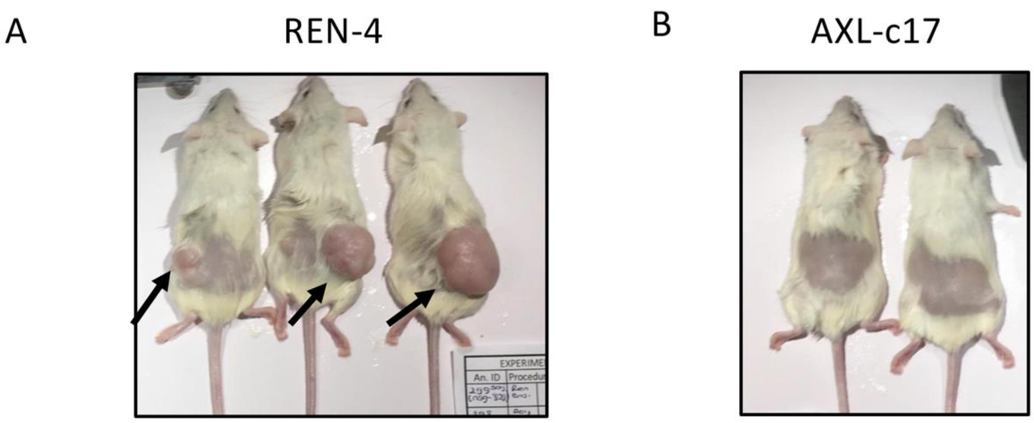

Figure S4. Examples of subcutaneous tumors formed by control REN-4 as compared to absence of tumors by AXL knock-out AXL-c17 cells in immunodeficient NOD-SCIDγ mice. Animals were shaved at cell injection sites to better visualize tumor formation.

Supplementary Methods:

β-Galactosidase Assay

β-Galactosidase Assay was performed as previously described. Briefly, non-treated and cells treated with 50 and 100 nM doxorubicin (Sigma, D15151) for 72 hours were fixed in 3% formaldehyde at room temperature. Cells were washed twice with 1xPBS. X-gal staining solution (ph=6.0) containing 40mM/ml citric acid, 140 mM/ml Na_2HPO_4, 60 mM/ml NaH_2PO_4, 5 mM/ml potassium ferricyanide and 5 mM/ml potassium ferrocyanide 150 mM/ml NaCl, 2 mM/ml MgCl2, and 1mg/ml X-gal) was added. Cells were incubated in 37°C for 16 hours. 10x Bright Field images were taken by Olympus IX71.
